# Propofol: monitoring for complications

**DOI:** 10.1186/cc13608

**Published:** 2014-03-17

**Authors:** F Yau, M Healy

**Affiliations:** 1 Royal London Hospital, London, UK

## Introduction

This re-audit aims to test our strategy for monitoring patients on propofol sedation for prolonged periods. Propofol infusion syndrome (PRIS), once established, is difficult to treat and currently there is limited guidance on how best to monitor for this potentially life-threatening complication [1]. An audit done in 2011 highlighted that lipid profile and electrocardiograms (ECGs) were rarely monitored. We recommended regular monitoring of these parameters when propofol sedation is used for over 3 days and that propofol-sparing agents are considered in these patients at risk of developing PRIS.

## Methods

In patients who required propofol sedation for 4 days or more, we prospectively monitored: frequency of performing lipid profile and 12-lead ECG; and frequency of co-administration of a propofol-sparing agent.

## Results

We collected data from 25 patients. The duration of propofol infusion was 4 to 15 days (mean 8.5 days). Mean propofol dose in the first 4 days was 2.2 mg/kg/hour. Maximum daily propofol dose ranged from 2.1 to 3.8 mg/kg/hour. Lipid profile was checked in 16/25 (64%) patients (20% in 2011) whilst on propofol sedation. However, these were checked following 3 days continuous infusion in only three patients. The triglyceride level was ≥2.2 mmol/l (very high) on first testing in 75% of patients. All patients had a 12-lead ECG done on admission. Seventeen of 25 (68%) had a further ECG performed whilst on continuous propofol infusion (45% in 2011). Additional ECGs were done in 13/17 patients (zero in 2011). ECG changes that we feel were attributable to propofol occurred in two patients, one of whom developed severe PRIS. Fourteen of 25 (56%) patients (55% in 2011) were on concomitant sedative agents. This included the patient who developed PRIS.

## Conclusion

Awareness of complications from prolonged propofol sedation is increasing amongst our clinicians. This is reflected in the increased frequency of lipid profile and ECG monitoring compared with 2011. However, we feel that more regular and routine monitoring is essential to aid early detection of this potentially fatal complication. We recommend that all patients at risk of developing PRIS should have these parameters monitored on a daily basis as standard.
